# Persistent Wnt/β-catenin signaling disables soft palatogenesis and palatal osteogenesis by inducing mesenchymal condensation

**DOI:** 10.3389/fcell.2026.1740081

**Published:** 2026-03-20

**Authors:** Biying Wang, Junyuan Xue, Yufan Bian, Jiamin Deng, Bo Liu, Nan Li, Lei Zhu, Jing Xiao, Chao Liu, Han Liu

**Affiliations:** 1 Department of Oral Pathology, Dalian Medical University School of Stomatology, Dalian, China; 2 Department of Periodontology and Oral Mucosal Diseases, General Hospital of Chinese People’s Liberal Army, Beijing, China; 3 Center of Stomatology, Xiangya Hospital of Central South University, Changsha, China; 4 Institute for Genome Engineered Animal Models of Human Diseases, Dalian Medical University, Dalian, China; 5 Dalian Key Laboratory of Basic Research in Oral Medicine, Dalian Medical University School of Stomatology, Dalian, China

**Keywords:** canonical Wnt signaling, cell migration, cleft palate, craniofacial osteogenesis, palatogenesis, soft palate, *Wnt5a*

## Abstract

**Introduction:**

Mammalian palates are composed of the anterior hard palate and the posterior soft palate. However, the correlation of the genesis, pattern formation, and morphogenesis between the hard and soft palates remains elusive.

**Methods:**

In this study, we explicated the complicated palatal defects in *Osr2-cre* ^*KI*^ *;Ctnnb1* ^*ex3f*^ mice, in which canonical Wnt activity was persistent due to constitutively active β-catenin in the palatal mesenchyme. *Osr2-cre* ^*KI*^ *;Ctnnb1* ^*ex3f*^ palates displayed an ectopic mesenchymal condensation extending from the proximal–posterior area to the distal–anterior area, along with impaired osteogenesis and agenesis of soft palate.

**Results:**

Immunohistochemistry showed the overlapping active canonical Wnt domain with the ectopic mesenchymal condensation, indicating that the condensation was induced by persistent canonical Wnt signaling. *Wnt5a*, a chemokine that induces posterior–anterior migration of palatal mesenchymal cells, was activated in the anterior and middle palatal mesenchyme of *Osr2-cre* ^*KI*^ *;Ctnnb1* ^*ex3f*^ mice. Exogenous supplementation of Wnt5a into wild-type (WT) palates recapitulated the mesenchymal condensation. These findings indicate that the persistent canonical Wnt signaling in the palatal mesenchyme extended *Wnt5a* expression, which enforced posterior mesenchymal migration toward the anterior to form the convoluted condensation, thereby impairing the genesis of the soft palate in *Osr2-cre* ^*KI*^ *;Ctnnb1* ^*ex3f*^ mice. Moreover, the medially osteogenic markers *Sox9*, *Runx2*, and *Osx*; the laterally *Shh*, *Foxf1*, and *Fgf10*; and another Wnt inhibitor, *Sfrp2*, were significantly reduced or even diminished in *Osr2-cre* ^*KI*^ *;Ctnnb1* ^*ex3f*^ palatal shelves. In contrast, the condensed *Osr2-cre* ^*KI*^ *;Ctnnb1* ^*ex3f*^ palatal mesenchyme displayed the medial markers Dlx5 and p-Smad1/5/8, along with the fibrosis/dermal markers ɑ-SMA and Tbx15. The Wnt and TGF-β/BMP inhibitors *Ectodin* and *Noggin* were also ectopically activated in the palatal epithelium overlying the condensed mesenchyme *Osr2-cre* ^*KI*^ *;Ctnnb1* ^*ex3f*^ mice.

**Discussion:**

These findings indicate a transition of palatal mesenchymal cells from an osteogenic fate into fibrosis commitment, along with disrupted mediolateral patterning of the palatal shelves due to persistent canonical Wnt activity. Our study provides molecular clues that fine-tuning the mesenchymal canonical Wnt activity and Wnt5a-directed cell migration correlates with the morphogenesis of hard palates and the genesis of soft palates.

## Introduction

The mammalian palate is composed of the anterior two-thirds hard palate, which is contributed by the primary palate and most of the secondary palate, and the posterior one-third soft palate, which originates from the posterior part of the secondary palate (Bush and Jiang, 2016). The hard palate contains the bony maxillary and palatine processes that physically separate the nasal and oral cavities, while the soft palate includes aponeurosis and paired muscles to not only create the nasal–oral separation but also allow speaking and swallowing (Li et al., 2019). The development of the hard palate includes the outgrowth of secondary palatal shelves from the lateral walls of the maxillary arches, the downward growth beside the tongue, horizontal re-orientation, contact and fusion with the contralateral palatal shelves, and osteogenic differentiation of the maxillary and palatine processes (Bush and Jiang, 2016; Li et al., 2017a). Unlike the dramatic morphogenesis in the secondary palatal shelves of hard palates, the development of soft palates is initiated from the most posterior secondary palatal shelves when the shelves are elevated horizontally. With the posterior extension, the shelves of the soft palates protrude horizontally from the lateral pharyngeal wall and grow to fuse with each other (Janečková et al., 2019; Li et al., 2019). Although the clear difference in histology makes the hard and soft palates appear as independent organs, the anatomy and temporal order of their development strongly indicate correlations in the genesis, pattern formation, and morphogenesis between the hard and soft palates. However, there are few studies on such a correlation during palatogenesis.

As one of the fundamental growth factors, Wnt ligands and canonical Wnt signaling were demonstrated to play essential roles in the development of secondary palates (Chen et al., 2009; He et al., 2011; Liu et al., 2015; Li et al., 2017b; Reynolds et al., 2019; Zhang et al., 2022). Previous studies exploiting Wnt reporter mouse lines indicated that the activity of canonical Wnt signaling was restricted to the palatal epithelium and excluded from the palatal mesenchyme (He et al., 2011). Both inactivation and constitutive activation of canonical Wnt signaling in the palatal epithelium resulted in cleft palates (He et al., 2011), indicating that fine-tuning of canonical Wnt signaling was critical for palatogenesis. However, the inactivation of canonical Wnt signaling in palatal mesenchyme by deleting β-catenin, the pivotal factor forming the trans-activator of canonical Wnt signaling with Tcf/Lef, also led to cleft palates (Chen et al., 2009; Janečková et al., 2023). Consistently, genotype and haplotype analyses in humans also indicated that mutations in Wnt genes were causative of cleft palates (Chiquet et al., 2008). Although cleft palates observed in loss-of-function mouse models indicated an indispensable role of the mesenchymal canonical Wnt signaling in palatogenesis, this conclusion was challenged by the fact that, in addition to mediating canonical Wnt signaling, β-catenin also contributes to cell behavior as a component of cytoskeleton (Salinas, 2007; Perez-Moreno and Fuchs, 2006). Thus, deleting β-catenin in the palatal mesenchyme was implied to cause cleft palates by disabling the cell cytoskeleton and migration. Therefore, the role of mesenchymal canonical Wnt signaling in palatogenesis still requires further exploration. On the other hand, although cleft palates caused by the activation of canonical Wnt signaling in palatal mesenchyme have been reported (Chen et al., 2009), the mechanism of how the elevated canonical Wnt activity disrupts palatal development has never been clarified. A recent study reported that the constitutive activation of canonical Wnt signaling in the palatal mesenchyme altered the expression pattern of genes involved in the cytoskeleton along the lateral–medial orientation (Wang et al., 2022), which implied that elevated canonical Wnt signaling caused cleft palates by disrupting cell migration. Furthermore, active Wnt signaling was also detected in the mesenchyme of soft palates, indicating a role of Wnt activity in soft palatogenesis (Janečková et al., 2019). A recent study on *Osr2-Cre;Ctnnb1* ^*f/f*^ mice indicated that canonical Wnt signaling regulated mesenchymal cell proliferation and subsequently myogenesis by mediating ciliogenesis during soft palatogenesis (Janečková et al., 2023). Thus, in our study, by crossing the *Osr2-cre* knock-in allele (*Osr2-cre* ^*KI*^) with the *Ctnnb1* ^*ex3f*^ knock-in allele, we activated canonical Wnt signaling constitutively in mouse palatal mesenchyme to examine how the elevated canonical Wnt activity impacts the hard and soft palatogenesis.

## Materials and methods

### Mouse lines

The *Osr2-cre* knock-in mouse (*Osr2-cre* ^*KI*^), *Shh-cre*, *Rosa26R-mT/mG*, *Ctnnb1* ^*ex3f*^, and *pMes-Noggin* mouse lines, which were obtained and described in previous studies (Wu et al., 2015; Li et al., 2021; Liu et al., 2022), were bred in the Specific Pathogen-Free System of the Institute of Genome-Engineered Animal Models for Human Diseases at Dalian Medical University. To constitutively activate Wnt signaling in the palatal mesenchyme, *Osr2-cre* ^*KI*^ mice were crossed with *Ctnnb1* ^*ex3f*^ mice to obtain *Osr2-cre* ^*KI*^ *;Ctnnb1* ^*ex3f*^ mouse embryos. The morning of the day of vaginal plug identification was regarded as embryonic day 0.5 (E 0.5). After inhaling carbon dioxide, timed-pregnant mice were euthanized through cervical dislocation. This research was approved by the Ethics Committee of Dalian Medical University (Protocol No. AEE18011), and all the animal experimental procedures strictly complied with the ethical guidelines of Dalian Medical University.

### Bulk RNA-seq analysis

Palates from E13.5 WT and *Osr2-cre* ^*KI*^ *;Ctnnb1* ^*ex3f*^ mice were dissected for RNA sequencing. The RNA-seq libraries were constructed according to our previously published protocol (Chen et al., 2025), with three biological replicates prepared for each sample. Differential expression analysis to identify differentially expressed genes (DEGs) between E13.5 WT and *Osr2-cre* ^*KI*^ *;Ctnnb1* ^*ex3f*^ palates was performed using the DESeq2 package, followed by Gene Ontology (GO) enrichment analysis of these DEGs using the clusterProfiler R package.

### Paraffin section and Masson’s trichrome staining

Embryonic mouse heads were collected from timed-pregnant mice in ice-cold phosphate-buffered solution (PBS) and fixed in 4% paraformaldehyde (PFA) overnight at 4 °C. After being dehydrated with gradient ethanol, staged heads were embedded in paraffin and consecutively sectioned at 10 μm for Masson’s trichrome staining, *in situ* hybridization, or immunohistochemistry, as described previously (Liu et al., 2022).

### Cell proliferation and apoptosis assays

For BrdU labeling, the timed-pregnant mice were intraperitoneally injected with BrdU labeling solution (10 mg/mL) at a dosage of 1 mL/100 g body weight. After an hour of observation, embryonic mouse heads were harvested and fixed in Carnoy’s fixative for 2 h. Following dehydration through an ethanol series, the heads were embedded in paraffin. Coronal sections were made at 10 µm and stained using the Detection Kit II (Roche Applied Science, Roche, Switzerland), following the manufacturer’s instructions. For the Ki67 immunofluorescence analysis, the antibody against Ki67 (1:2,000; Abcam, ab15580) was applied with the secondary antibody in the MaxVision TM HRP Polymer anti-Mouse/Rabbit IHC Kit (No. KIT5020, Maixin Ltd., Fuzhou, China). The TUNEL labeling assay was performed to detect cell apoptosis using the *In Situ* Cell Death Detection Kit, POD (No. 11684817910, Roche).

### Cryostat section and phalloidin staining

After being fixed in a mixture containing 4% PFA and 15% sucrose overnight at 4 °C, embryonic mouse heads of the selected stages were immersed in 30% sucrose and embedded in O.C.T. compound (Tissue-Tek, Sakura®Finetek, VWR, Torrance, CA, United States) for coronal sections at 10 µm. For phalloidin (ab176753, Abcam, Cambridge, MA, United States) staining, cryo-sections were permeabilized for 1 h in 0.3% Triton-X 100 at room temperature prior to incubation with the phalloidin conjugate reagent for 1 h. Sections were counterstained with DAPI and imaged under an Olympus DP72 microscope.

### *In situ* hybridization

Embryos were dissected in diethyl pyrocarbonate (DEPC)-treated PBS and fixed with 4% PFA in 0.1% DEPC-treated PBS overnight at 4 °C. The fixed heads were dehydrated through graded ethanol, embedded in paraffin, and sectioned at 10 µm for *in situ* hybridization (ISH). For whole-mount ISH, fixed heads were also dehydrated through a methanol series. All sense and anti-sense RNA probes were transcribed from the plasmids containing the mouse-linearized cDNA templates using an RNA Labeling Kit (Roche, Indianapolis, IN, United States), as described previously (Liu et al., 2022). The alkaline phosphatase-conjugated anti-digoxigenin (DIG) antibody was used to incubate whole mounts or sections. The hybridization signal was detected by BM Purple (Roche, Indianapolis, IN, United States). Nuclear fast red was used for counter-staining in all sections.

### Immunohistochemistry and immunofluorescence

Immunohistochemistry and immunofluorescence were carried out on the 10-µm-thick paraffin sections. The primary antibodies against Lef1 (ab137827, Abcam, Cambridge, MA, United States), integrin ɑv (bs-1310R, Bioss Antibodies Inc., Beijing, China), ColII (K0921, Santa Cruz), Foxf1 (ab308633, Abcam), Dlx5 (10592-1-AP, Proteintech Group Inc., Wuhan, China), Runx2 (sc-101145, Santa Cruz Biotechnology Co., Ltd., Shanghai, China), Sox9 (ab185966, Abcam), Osterix (ab209484, Abcam), phospho-Smad1/5/8 (13820S, Cell Signaling Technology, Danvers, MA, United States), ɑ-SMA (ab7817, Abcam), ColI (P28372-BIF, Abmart, Shanghai, China), p-ERK1/2 (4370T, Cell Signaling Technology), Etv4 (10684-1-AP, Cloud-Clone Corp., Wuhan, China), Tbx15 (YN1399, Immunoway, Thermo Fisher Scientific, Shanghai, China), and Scx (ab307722, Abcam) were applied for immunofluorescence. The TSA Fluorescence Triple Staining Kit (G1236-100T, ServiceBio Co. Ltd., Wuhan, China) was applied for fluorescence development using the secondary antibody of IF Tyramide (1:1,000; ServiceBio, G1236-50T), showing green fluorescence at a wavelength of 488 nm and red fluorescence at 647 nm. The sections were counterstained with DAPI.

### Agarose bead implantation and organ culture

The recombinant mouse Wnt5a protein (URPP549Mu01, Cloud-Clone Corp., Wuhan, China) was dissolved in phosphate-buffered saline at 0.5 mg/mL. The agarose beads (1537302, Bio-Rad) were incubated with the Wnt5a working solution for 1 hour prior to implantation into the E13.5 mouse palatal shelves. After 12 h of organ culture in Trowell dishes, the palatal shelves were fixed and sectioned for immunofluorescence staining with an antibody against Tbx15. The implantation was performed three times with different litters.

### Statistical assay

For cell density, cell proliferation, and apoptosis, the Ki67-positive, BrdU-positive, TUNEL-positive, and total nuclei in bilateral palatal shelves were counted using ImageJ (version 1.54 g). For measuring the color intensity in ISH and the percentages of active domains to the entire palatal shelves in immunofluorescence, the images were converted to 8-bit grayscale with ImageJ, in which the color/fluorescence from the adjacent unstained or fluorescence-free areas was set as the background. Three independent replicates were performed in the paired WT and *Osr2-cre* ^*KI*^ *;Ctnnb1* ^*ex3f*^ littermates. A two-tailed Student’s t-test was conducted using GraphPad Prism 9 (GraphPad Software Inc., United States) and presented as the mean with standard deviation (SD), with statistical significance set at *p* < 0.05.

## Results

### *Osr2-cre* ^*KI*^ *;Ctnnb1* ^*ex3f*^ mice displayed ectopic condensed mesenchyme in the palatal shelves

**FIGURE 1:**
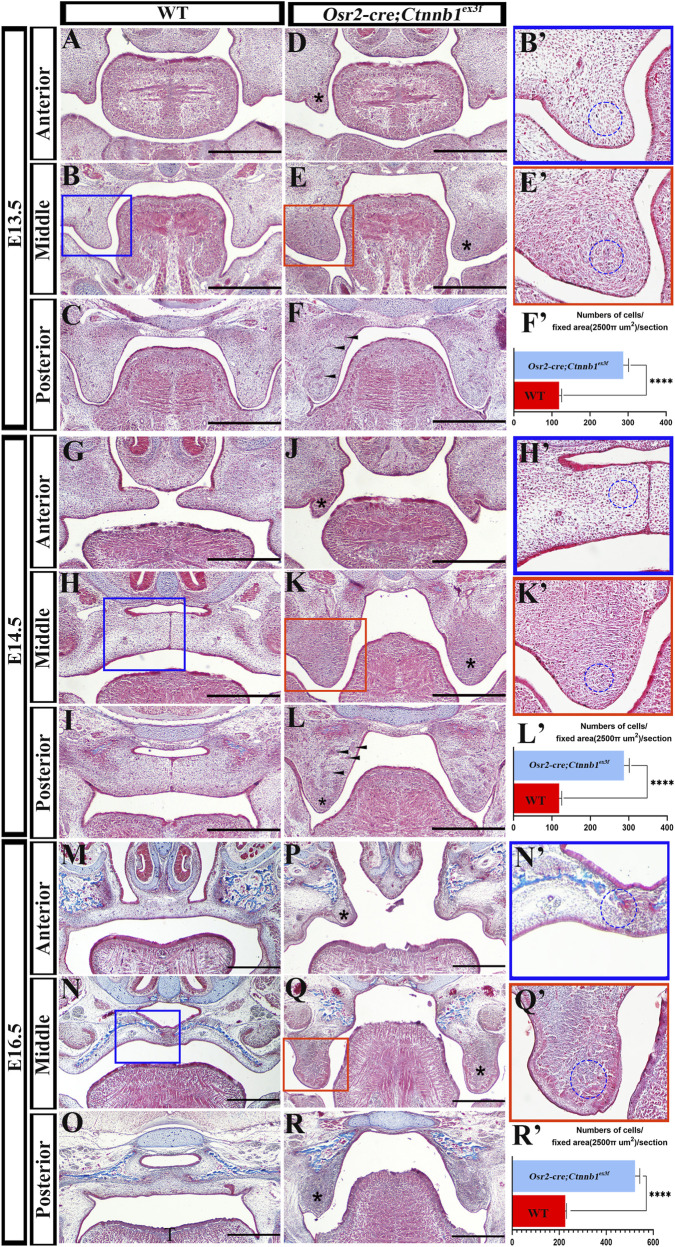
Masson staining of the hard palates in *Osr2-cre* ^*KI*^ *; Ctnnb1* ^*ex3f*^ mice. Masson staining showed the histology of E13.5 WT hard palatal shelves **(A–C)** and E13.5 *Osr2-cre* ^*KI*^ *;Ctnnb1* ^*ex3f*^ hard palatal shelves **(D–F)**; **(B′,E′)** the corresponding boxed areas in **(B,E)**; **(F′)** statistical assay showed the increased cell density in *Osr2-cre* ^*KI*^ *;Ctnnb1* ^*ex3f*^ palatal shelves compared to that in WT counterparts (WT: 118.7 ± 6.81/2500 π μm^2^ vs. *Osr2-cre* ^*KI*^ *; Ctnnb1* ^*ex3f*^: 287.3 ± 14.05/2500 π μm^2^, *p* < 0.001). **(G–I)** Histology of the E14.5 WT hard palate; **(J–L)** histology of E14.5 *Osr2-cre* ^*KI*^ *;Ctnnb1* ^*ex3f*^ hard palatal shelves; **(H′,K′)** the corresponding boxed areas in **(H,K)**; **(L′)** statistical assay showed the increased cell density in *Osr2-cre* ^*KI*^ *;Ctnnb1* ^*ex3f*^ palatal shelves compared to that in WT counterparts (WT: 138.0 ± 6.25/2500 π μm^2^ vs. *Osr2-cre* ^*KI*^ *; Ctnnb1* ^*ex3f*^: 335.0 ± 5.08/2500 π μm^2^, *p* < 0.001). **(M–O)** Histology of the E16.5 WT hard palate; **(P,O,R)** histology of E16.5 *Osr2-cre* ^*KI*^ *;Ctnnb1* ^*ex3f*^ hard palatal shelves; **(N′,Q′)** the corresponding boxed areas in **(N,Q)**; **(R′)** statistical assay showed the increased cell density in *Osr2-cre* ^*KI*^ *;Ctnnb1* ^*ex3f*^ palatal shelves compared to that in WT counterparts (WT: 225.3 ± 5.03/2500 π μm^2^ vs. *Osr2-cre* ^*KI*^ *; Ctnnb1* ^*ex3f*^: 522.3 ± 20.06/2500 π μm^2^, *p* < 0.001). (The dashed blue circles in **(B′,E′,H′,K′,N′,Q′)** show the fixed areas for cell counting; asterisks and arrowheads indicate the condensed mesenchyme in *Osr2-cre* ^*KI*^ *;Ctnnb1* ^*ex3f*^ hard palatal shelves; scale bars: 500 μm; ****, *p* < 0.0001).

Cryostat sections of *Osr2-cre* ^*KI*^ *;Rosa26R-mT/mG* mice showed that the Cre activity was only detected in the mesenchyme of hard and soft palates but was absent from the palatal epithelium (Supplementary Figure S1). To explore how constitutively activated canonical Wnt signaling in the palatal mesenchyme caused cleft palates, we first explicated the histological characteristics of *Osr2-cre* ^*KI*^ *;Ctnnb1* ^*ex3f*^ palates. Masson’s trichrome staining showed that compared to the anterior, middle, and posterior areas of the E13.5 palatal shelves (Figures 1A–C), the *Osr2-cre* ^*KI*^ *;Ctnb1* ^*ex3f*^ palatal shelves were enlarged (Figures 1D–F), with visible mesenchymal condensation (Figures 1B′,E′,F′). At E14.5, when the WT palate shelves were reoriented horizontally and even contacted at the middle and posterior levels (Figures 1G–I), the *Osr2-cre* ^*KI*^ *;Ctnnb1* ^*ex3f*^ palate shelves were still vertically beside the tongue (Figures 1J–L), with the more evident mesenchymal condensation through the palatal shelves (Figures 1H′,K′,L′). In contrast to the fused E16.5 WT palates with noticeably ossifying centers in the middle and posterior areas (Figures 1M,N,Q), the enlarged *Osr2-cre* ^*KI*^ *;Ctnnb1* ^*ex3f*^ palate shelves became more severe (Figures 1P,Q,R), with the mesenchymal condensation almost throughout the distal anterior, middle, and posterior shelves (Figures 1N′,Q′,R′). These findings indicated an association of the constitutively active canonical Wnt signaling in palatal mesenchyme with ectopic mesenchymal condensation.

### The enhanced cell adhesion convoluted cell arrangement but reduced cell proliferation in the condensed *Osr2-cre* ^*KI*^ *;Ctnnb1* ^*ex3f*^ palatal mesenchyme

**FIGURE 2:**
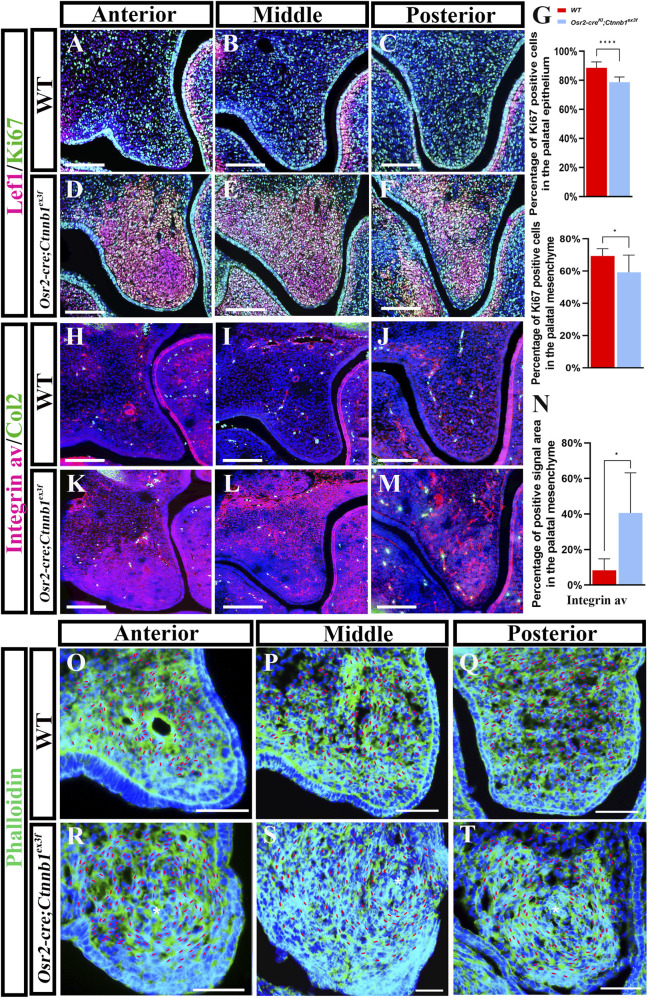
Canonical Wnt signaling distribution, cell proliferation, adhesion, and arrangement in *Osr2-cre* ^*KI*^ *;Ctnnb1* ^*ex3f*^ palatal shelves. **(A–F)** Immunofluorescence staining of Lef1 and Ki67 in E13.5 WT palatal shelves **(A–C)** and E13.5 *Osr2-cre* ^*KI*^ *;Ctnnb1* ^*ex3f*^ mice **(D–F)**. **(G)** Ki67 positive percentages reduced from 88.64% ± 4.04% and 69.38% ± 4.51% in WT to 78.67 ± 3.59 (*p* < 0.0001) and 59.26 ± 10.66 (*p* < 0.05) in the *Osr2-cre* ^*KI*^ *;Ctnnb1* ^*ex3f*^ palatal epithelium and mesenchyme, respectively. **(H–M)** Immunofluorescence staining of integrin ɑv and ColII in E13.5 WT palatal shelves **(H–J)** and E13.5 *Osr2-cre* ^*KI*^ *;Ctnnb1* ^*ex3f*^ mice **(K–M)**. **(N)** The percentage of integrin ɑv-positive areas increased from 8.30% ± 6.48% in WT to 40.64% ± 22.50% (*p* < 0.05) in *Osr2-cre* ^*KI*^ *;Ctnnb1* ^*ex3f*^ palatal shelves. **(O–T)** Immunofluorescence of phalloidin showed the cytoskeletal orientation in the E13.5 WT palate shelves **(O–Q)** and the E13.5 *Osr2-cre* ^*KI*^ *;Ctnnb1* ^*ex3f*^ palate shelves **(R–T)**. (Asterisks in **(R–T)** represent the centers of the convoluted mesenchymal loops; red short lines in **(O–T)** delineated the longitudinal axes of mesenchymal cells; *, *p* < 0.05; ****, *p* < 0.0001; scale bars: 100 μm).

To reveal the correlation between the activated canonical Wnt signaling and mesenchymal condensation, immunohistochemical staining of Lef1, a nuclear mediator of canonical Wnt signaling, was performed. In E13.5 WT palates, Lef1 staining was only detected in a few mesenchymal and epithelial cells of the anterior (Figure 2A) and middle shelves (Figure 2B), but it was absent from the posterior palatal shelves (Figure 2C). In contrast, the Lef1-positive cells in E13.5 *Osr2-cre* ^*KI*^ *;Ctnnb1* ^*ex3f*^ palatal shelves were not only absent from palatal epithelium but also coincided with the location of the condensed mesenchyme in the anterior (Figure 2D), middle (Figure 2E), and posterior (Figure 2F) shelves, which strongly indicated the induction of mesenchymal condensation by the active canonical Wnt signaling. Ki67 staining indicated the remarkably reduced proliferation in both the condensed mesenchymal and epithelial cells of *Osr2-cre* ^*KI*^ *;Ctnnb1* ^*ex3f*^ palatal shelves (Figures 2D–G) compared to that in the WT counterparts (Figures 2A–C,G). Moreover, BrdU labeling assays indicated that the tip of the E13.5 anterior *Osr2-cre* ^*KI*^ *;Ctnnb1* ^*ex3f*^ palatal shelves, which was not reached by mesenchymal condensation, exhibited more BrdU-positive nuclei than the WT control (Supplementary Figures S2A,B). In contrast, there were fewer BrdU-positive nuclei in the condensed *Osr2-cre* ^*KI*^ *;Ctnnb1* ^*ex3f*^ anterior–mid palatal shelves than in the WT counterparts (Supplementary Figures S2C,D). These phenomena indicated that the proliferating cells were pushed into the most anterior tip of the palates by the condensed cells, which increased the proliferation ratio in the anterior tip. On the other hand, the TUNEL assay showed no difference in cell apoptosis between WT and *Osr2-cre* ^*KI*^ *;Ctnnb1* ^*ex3f*^ palatal shelves (Supplementary Figures S2E–J). Therefore, the active canonical Wnt signaling most likely enhanced the condensation but reduced cell proliferation in the palatal mesenchyme.

Furthermore, integrin ɑv staining in the mesenchymal condensation of E13.5 *Osr2-cre* ^*KI*^ *; Ctnnb1* ^*ex3f*^ palatal shelves was more extensive and robust (Figures 2K–N) than that in WT controls (Figures 2H–J), indicating enhanced cell adhesion in the condensed mesenchymal cells. Both the cytoskeletal F-actin marked by phalloidin and nuclei orientation by DAPI staining indicated a convoluted loop with a center of condensed mesenchymal cells in the anterior (Figure 2R), middle (Figure 2S), and posterior (Figure 2T) of the *Osr2-cre* ^*KI*^ *;Ctnnb1* ^*ex3f*^ palatal shelves compared to the distal–proximal and lateral–medial arrangement in the WT counterparts (Figures 2O,P,Q). Taken together, this indicates that persistently active canonical Wnt signaling in the palatal mesenchyme most likely induced mesenchymal adhesion that condensed cells into a convoluted loop but reduced proliferation.

### Ectopic *Wnt5a* activation and diminished *Sfrp2* expression in *Osr2-cre* ^*KI*^ *;Ctnnb1* ^*ex3f*^ palatal shelves

**FIGURE 3:**
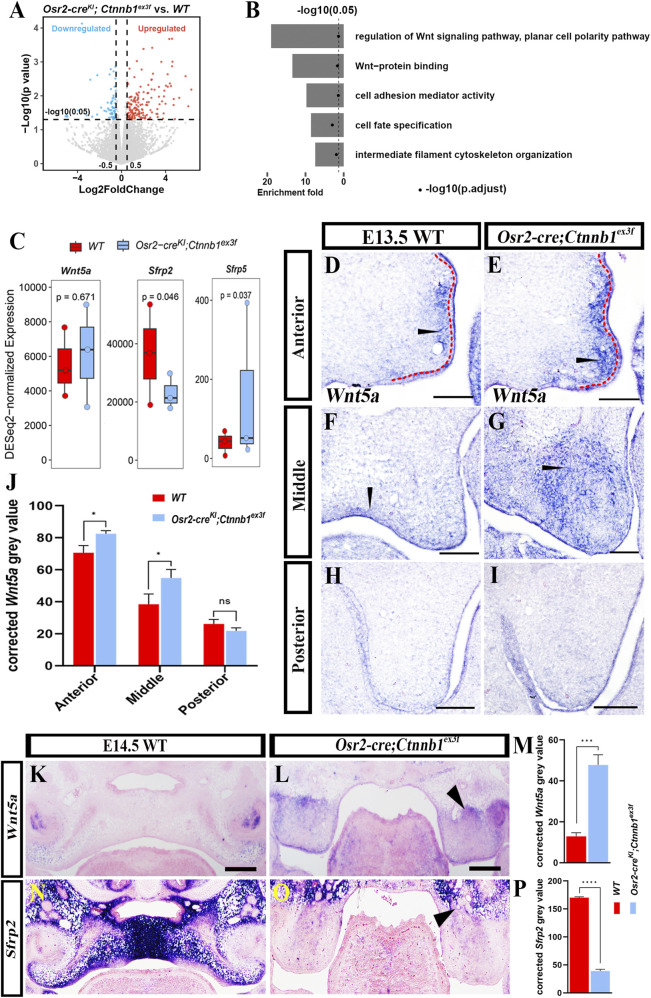
Suppressed *Sfrp2* and enhanced *Wnt5a* expression in *Osr2-cre* ^*KI*^ *; Ctnnb1* ^*ex3f*^ palatal shelves. **(A)** Volcano plot visualization of DEGs between E13.5 WT and *Osr2-cre* ^*KI*^ *;Ctnnb1* ^*ex3f*^ palates. **(B)** GO enrichment analysis of DEGs between E13.5 WT and *Osr2-cre* ^*KI*^ *;Ctnnb1* ^*ex3f*^ palates, as presented in **(A)**. The length of each horizontal bar corresponds to the enrichment fold. The dot paired with each bar represents −log10 (*p*.adjust) (negative logarithm of the adjusted *p*-value). The dashed vertical line denotes the significance threshold (−log10 (0.05)), and all terms presented in this plot satisfy the criterion of *p*. adjust <0.05. **(C)** Boxplots comparing DESeq2-normalized expression of *Wnt5a*, *Sfrp2*, and *Sfrp5* between E13.5 WT and *Osr2-cre* ^*KI*^ *;Ctnnb1* ^*ex3f*^ palates, with statistical significance evaluated using the Wald test in the DESeq2 package. **(D–I)** *In situ* hybridization of *Wnt5a* in E13.5 WT anterior **(D)**, middle **(F)**, and posterior **(H)** palatal shelves and in E13.5 *Osr2-cre* ^*KI*^ *;Ctnnb1* ^*ex3f*^ **(E)** middle **(G)** and posterior **(I)** palatal shelves. **(J)** Statistical assay showed the normalized signal intensity of *Wnt5a* transcription **(D–I)**. **(K,L)** *In situ* hybridization of *Wnt5a* in E14.5 WT **(K)** and *Osr2-cre* ^*KI*^ *;Ctnnb1* ^*ex3f*^ middle palates **(L)**. **(M)** Statistical assay of *Wnt5a* transcription normalized from **(K,L)**. **(N,O)** *In situ* hybridization of *Sfrp2* in E14.5 WT **(N)** and *Osr2-cre* ^*KI*^ *;Ctnnb1* ^*ex3f*^ middle palates **(O)**. **(P)** Statistical assay of *Sfrp2* transcription normalized from **(N,O)**. (Black arrowheads in **(D–G)** indicate the signals; scale bars: 100 μm; *, *p* < 0.05; ***, *p* < 0.001; ****, *p* < 0.0001).

To explicate the alteration in the gene expression profile of palatal mesenchymal cells, the E13.5 WT and *Osr2-cre* ^*KI*^ *;Ctnnb1* ^*ex3f*^ palatal shelves were collected for bulk RNA-seq. The DEGs were read out using the DESeq2 package (Figure 3A; Supplementary Material 1). Furthermore, GO analysis enriched the DEGs in regulation in the Wnt signaling pathway/planar cellular polarity pathway, Wnt-protein binding, cell adhesion-mediator activity, cell fate specification, and intermediate filament cytoskeleton organization (Figure 3B; Supplementary Material 2). Bulk RNA-seq showed significant downregulation of *Sfrp2* and upregulation of *Sfrp5* expression, but no difference was observed in *Wnt5a* transcription between *Osr2-cre* ^*KI*^ *;Ctnnb1* ^*ex3f*^ and WT control palates (Figure 3C). However, *in situ* hybridization revealed that *Wnt5a* expression, which was detected in the anterior and middle lateral mesenchyme of E13.5 WT palatal shelves (Figures 3D,F,H), was significantly enhanced in the anterior and even extended throughout the middle mesenchyme of *Osr2-cre* ^*KI*^ *;Ctnnb1* ^*ex3f*^ palatal shelves (Figures 3E,G,I,J). The *in situ* hybridization in E14.5 WT and *Osr2-cre* ^*KI*^ *;Ctnnb1* ^*ex3f*^ palates confirmed the ectopic activation of *Wnt5a* (Figures 3K–M), along with the diminished *Sfrp2* expression in *Osr2-cre* ^*KI*^ *;Ctnnb1* ^*ex3f*^ middle palatal shelves (Figures 3N–P).

### The Wnt5a-induced cell migration and condensation led to the agenesis of the soft palate in *Osr2-cre* ^*KI*^ *;Ctnnb1* ^*ex3f*^ mice

**FIGURE 4:**
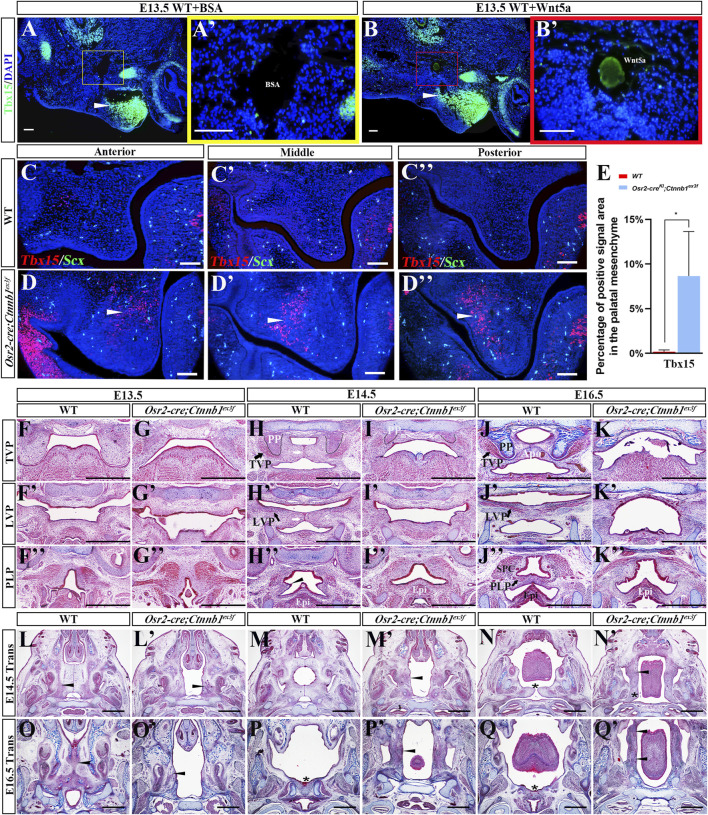
Wnt5a-induced cell migration and condensation impaired the genesis of *Osr2-cre* ^*KI*^ *;Ctnnb1* ^*ex3f*^ soft palates. **(A,B,A′,B′)** Sagittal views showing Tbx15 immunofluorescence staining in E13.5 WT palatal shelves supplemented with BSA **(A)** or exogenous Wnt5a **(B)**. **(A′,B′)** Corresponding amplified boxed areas in **(A,B)**. **(C,D,C′,D′,C′′,D′′)** Cross-sections displaying the immunofluorescence of Tbx15 and Scx in E13.5 WT soft palates **(C,C′,C′′)** and E13.5 *Osr2-cre* ^*KI*^ *; Ctnnb1* ^*ex3f*^ soft palatal shelves **(D,D′,D′′)**. **(E)** Percentage of the Tbx15-positive areas to entire palatal shelves increased from 0.18% ± 0.2% in WT to 8.66% ± 4.99% (*p* < 0.05) in *Osr2-cre* ^*KI*^ *;Ctnnb1* ^*ex3f*^ mice. **(F–Q,F′–Q′,F′′–Q′′)** Cross and transverse histology features of *Osr2-cre* ^*KI*^ *;Ctnnb1* ^*ex3f*^ soft palates. Cross-sections of E13.5 WT soft palates **(F,F′,F′′)** and E13.5 *Osr2-cre* ^*KI*^ *;Ctnnb1* ^*ex3f*^ soft palatal shelves **(G,G′,G′′)**. Cross-sections of E14.5 WT soft palates **(H,H′,H′′)** and E14.5 *Osr2-cre* ^*KI*^ *;Ctnnb1* ^*ex3f*^ soft palatal shelves **(I,I′,I′′)**. Cross-sections of E16.5 WT soft palates **(J,J′,J′′)** and E16.5 *Osr2-cre* ^*KI*^ *;Ctnnb1* ^*ex3f*^ soft palatal shelves **(K,K′,K′′)**. Transverse sections of E14.5 WT **(L–N)** and *Osr2-cre* ^*KI*^ *;Ctnnb1* ^*ex3f*^ hard and soft palatal shelves **(L′–N′)**. Transverse sections of E16.5 WT **(O–Q)** and *Osr2-cre* ^*KI*^ *;Ctnnb1* ^*ex3f*^ hard and soft palatal shelves **(O′,P′,Q′)**. (White arrowheads in **(A,B)** indicate the anterior margin of Tbx15 domains; white arrowheads in **(D,D′,D′′)** indicate the Tbx15 positive cells; TVP, tensor veli palatini; LVP, levator veli palatini; PLP, palatopharyngeus; PP, pterygoid process; SPC, superior pharyngeal constrictor; Epi, epiglottis; Apo, aponeurosis. The dashed lines in **(H,I)** delineate PP; black arrows indicate TVP, LVP, and PP; black arrowheads point to the condensed mesenchyme in *Osr2-cre* ^*KI*^ *;Ctnnb1* ^*ex3f*^ palatal shelves; asterisks indicate soft palate; *, *p* < 0.05; scale bars in **(A–D)**: 100 μm; scale bars in **(F–Q)**: 500 μm).

To examine whether the ectopic *Wnt5a* activation played a role in palatal mesenchymal condensation, Wnt5a-soaked agarose beads were grafted into the E13.5 WT middle–posterior palatal shelves for organ culture. The sagittal sections of the cultured palatal shelves implicated a remarkable cell condensation around the Wnt5a-soaked beads (Figures 4B,B′) instead of around the BSA-soaked beads (Figures 4A,A′). Further immunofluorescence staining displayed an anterior extension of Tbx15 expression, a dermal marker in the soft palate, to Wnt5a-soaked beads from the most posterior palatal shelves (Figures 4B,B′) compared to BSA beads (Figures 4A,A′), indicating that the supplemented Wnt5a chemo-attracted the mesenchymal cells to migrate anteriorly and form the condensation. Consistently, the cross-sections in E13.5 *Osr2-cre* ^*KI*^ *;Ctnnb1* ^*ex3f*^ palatal shelves exhibited the ectopic Tbx15 cells in the mesenchymal condensation (Figures 4C,C′,C″,D,D′,D″,E), indicating that ectopic Wnt5a was likely important for the migration of posterior mesenchymal cells toward the anterior.

We further assessed the impact of the ectopic *Wnt5a* activation and mesenchymal condensation on palatogenesis and found the agenesis of soft palates in *Osr2-cre* ^*KI*^ *;Ctnnb1* ^*ex3f*^ mice. The cross-sections showed that the soft palatal shelves were downward at the tensor veli palatini (TVP) level, horizontally protruded at the levator veli palatini (LVP) level, and absent at the palatopharyngeus (PLP) level in E13.5 WT mice (Figures 4F,F′,F″). In contrast, the soft palatal shelves were completely absent in the TVP, LVP, and PLP levels of E13.5 *Osr2-cre* ^*KI*^ *;Ctnnb1* ^*ex3f*^ mice (Figures 4G,G′,G″). When the soft palatal shelves contacted and even fused at the TVP and LVP levels and protruded at the PLP level in E14.5 WT mice (Figures 4H,H′,H″), there were no soft palatal shelves protruding from the maxillary and pharyngeal walls in *Osr2-cre* ^*KI*^ *;Ctnnb1* ^*ex3f*^ mice (Figures 4I,I′,I″). At E16.5, the TVP, LVP, and PLP were notable in the WT soft palates (Figures 4J,J′,J″), while *Osr2-cre* ^*KI*^ *;Ctnnb1* ^*ex3f*^ mice exhibited not only the absence of TVP, LVP, and PLP but also penetrated oral and nasal cavities (Figures 4K,K′,K″). These consequences indicated the agenesis of soft palates in *Osr2-cre* ^*KI*^ *;Ctnnb1* ^*ex3f*^ mice. Furthermore, transverse sections were performed to further reveal the mesenchymal condensation of *Osr2-cre* ^*KI*^ *;Ctnnb1* ^*ex3f*^ palatal shelves. At the top level of E14.5 maxillary molars, the fusing hard palatal shelves of WT mice showed the condensed maxillary and palatine mesenchyme (Figure 4L), while the *Osr2-cre* ^*KI*^ *;Ctnnb1* ^*ex3f*^ mice displayed sporadic condensation in the posterior palatal shelves (Figure 4L′). At the medial level of E14.5 maxillary molars, the mesenchymal condensation was excluded in WT hard palates but was detected around the pterygoid processes (PP) in soft palates (Figure 4M). In contrast, both the posterior hard palatal shelves and the mesenchyme surrounding PP in *Osr2-cre* ^*KI*^ *;Ctnnb1* ^*ex3f*^ mice were occupied by the condensation (Figure 4M′). At the bottom level of E14.5 maxillary molars, few mesenchymal condensation were found in WT soft palates (Figure 4N), while the enlarged distal palatal shelves in *Osr2-cre* ^*KI*^ *;Ctnnb1* ^*ex3f*^ mice displayed condensations located in the anterior and middle shelves but absent in the posterior palatal shelves (Figure 4N′). Similarly, at the top level of E16.5 maxillary molars, both the WT and *Osr2-cre* ^*KI*^ *;Ctnnb1* ^*ex3f*^ palates phenocopied their E14.5 counterparts (Figures 4O,O′). At the median and bottom levels of E16.5 maxillary molars, the PP, aponeurosis, and PLP were notable in WT soft palates without the condensed mesenchyme (Figures 4P,Q). In contrast, the condensation was extended throughout the *Osr2-cre* ^*KI*^ *;Ctnnb1* ^*ex3f*^ palatal shelves (Figures 4P′,Q′). Thus, the mesenchymal condensation was extended from top (proximal)–posterior to bottom (distal)–anterior in the *Osr2-cre* ^*KI*^ *;Ctnnb1* ^*ex3f*^ palatal shelves.

### The condensed *Osr2-cre* ^*KI*^ *;Ctnnb1* ^*ex3f*^ palatal mesenchyme was deprived of osteogenic capacity

**FIGURE 5:**
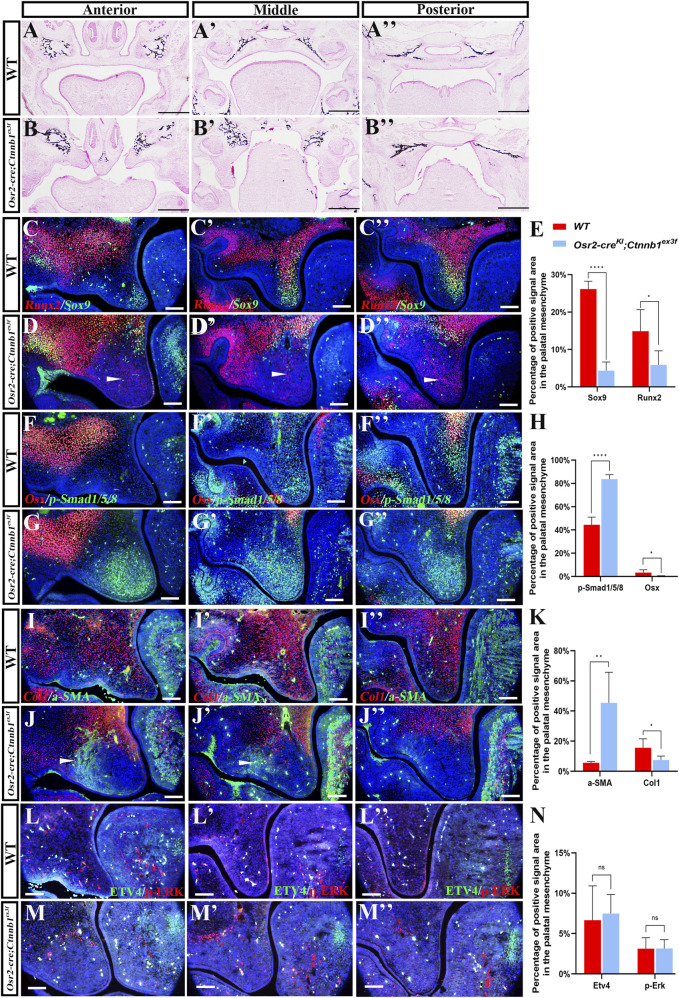
Impaired osteogenesis in the condensed *Osr2-cre* ^*KI*^ *;Ctnnb1* ^*ex3f*^ palatal mesenchyme. **(A,A′,A′′,B,B′,B′′)** von Kossa staining displayed the ossified maxillary and palatine bones in the E16.5 WT hard palate **(A,A′,A′)** and *Osr2-cre* ^*KI*^ *;Ctnnb1* ^*ex3f*^ hard palatal shelves **(B,B′,B′)**. **(C,C′,C′′,D,D′,D′′)** Immunofluorescence of Runx2 and Sox9 in E13.5 WT anterior **(C)**, middle **(C′)**, and posterior **(C′′)** palatal shelves and in E13.5 *Osr2-cre* ^*KI*^ *;Ctnnb1* ^*ex3f*^ **(D)** middle **(D′)** and posterior **(D′′)** palatal shelves. **(E)** The percentages of Runx2- and Sox9-positive areas to the entire palatal shelves decreased from 14.88% ± 5.80% and 26.15% ± 2.08% in WT to 5.89% ± 3.75% (*p* < 0.05) and 4.35% ± 2.35% (*p* < 0.0001) in *Osr2-cre* ^*KI*^ *;Ctnnb1* ^*ex3f*^ mice, respectively. **(F,F′,F′′,G,G′,G′′)** Immunofluorescence of Osx and p-Smad1/5/8 in the anterior **(F)**, middle **(F′)**, and posterior **(F′′)** palatal shelves of E13.5 WT mice and the anterior **(G)**, middle **(G′)**, and posterior **(G′′)** palatal shelves of E13.5 *Osr2-cre* ^*KI*^ *; Ctnnb1* ^*ex3f*^ mice. **(H)** The percentage of the Osx-positive area to the entire palatal shelves decreased from 3.37% ± 2.45% in WT to 5.89% ± 3.75% (*p* < 0.05) and 0.31% ± 0.42% (*p* < 0.05) in *Osr2-cre* ^*KI*^ *;Ctnnb1* ^*ex3f*^ mice, while the percentage of the p-Smad1/5/8-positive area to the entire palatal shelves increased from 4.35% ± 2.35% in WT to 26.15% ± 2.08% (*p* < 0.0001) in *Osr2-cre* ^*KI*^ *;Ctnnb1* ^*ex3f*^ mice. **(I,I′,I′′,J,J′,J′′)** Immunofluorescence of Col1 and ɑ-SMA in the anterior **(I)**, middle **(I′)**, and posterior **(I′′)** palatal shelves of E13.5 WT mice and the anterior **(J)**, middle **(J′)**, and posterior **(J′′)** palatal shelves of E13.5 *Osr2-cre* ^*KI*^ *; Ctnnb1* ^*ex3f*^ mice. **(K)** The percentage of the ColI-positive area to the entire palatal shelves decreased from 15.67% ± 5.90% in WT to 7.51% ± 2.62% (*p* < 0.01) in *Osr2-cre* ^*KI*^ *;Ctnnb1* ^*ex3f*^ mice, while the percentage of the a-SMA-positive area to the entire palatal shelves increased from 5.64% ± 0.92% in WT to 45.43% ± 20.31% (*p* < 0.05) in *Osr2-cre* ^*KI*^ *;Ctnnb1* ^*ex3f*^ mice. **(L,L′,L′′,M,M′,M′′)** Immunofluorescence of p-Erk1/2 and Etv4 in the anterior **(L)**, middle **(L′)**, and posterior **(L′′)** palatal shelves of E13.5 WT mice and the anterior **(M)**, middle **(M′)**, and posterior **(M′′)** palatal shelves of E13.5 *Osr2-cre* ^*KI*^ *;Ctnnb1* ^*ex3f*^ mice. **(N)** The percentages of the p-Erk1/2- and Etv4-positive areas to the entire palatal shelves were slightly different between WT (3.13% ± 1.35% and 6.65% ± 4.25%) and *Osr2-cre* ^*KI*^ *;Ctnnb1* ^*ex3f*^ mice (7.48% ± 2.38%, *p* > 0.05 and 3.15% ± 1.10%, *p* > 0.05), respectively. (White arrowheads point to the immunofluorescence signal in palatal shelves; *, *p* < 0.05; **, *p* < 0.01; ****, *p* < 0.0001; scale bars in **(A,B)** 500 μm; scale bars in **(C–M)** 100 μm).

The von Kossa staining showed that the ossified bones were extending to the secondary ossification centers in the E16.5 distal WT palate (Figures 5A,A′,A″) but were absent in the mesenchymal condensation of the *Osr2-cre* ^*KI*^ *;Ctnnb1* ^*ex3f*^ palate shelves (Figures 5B,B′,B″), indicating an impaired palatal osteogenesis correlated to the constitutively active canonical Wnt signaling in the palatal mesenchyme. To further address the molecular characteristics of the condensed mesenchymal cells in *Osr2-cre* ^*KI*^ *;Ctnnb1* ^*ex3f*^ palatal shelves, the expression pattern of several osteogenic markers was first checked by immunofluorescence staining. The Sox9 staining was detected in the distal tip of the anterior and the medial side of the middle and posterior shelves in E13.5 WT palatal mesenchyme (Figures 5C,C′,C″) but was almost completely absent in the *Osr2-cre* ^*KI*^ *;Ctnnb1* ^*ex3f*^ palatal mesenchyme (Figures 5D,D′,D″,E). Similarly, the osteogenic marker Runx2, which was located in the medial mesenchyme of the middle and posterior E13.5 WT palatal shelves (Figures 5C,C′), was only sporadically detected in the condensed mesenchyme of *Osr2-cre* ^*KI*^ *;Ctnnb1* ^*ex3f*^ palatal shelves without extending into the distal side of the medial mesenchyme (Figures 5D,D′,D″,E). Another osteogenic marker, Osx, which displayed a similar but reduced domain as Runx2 did in E13.5 WT palatal shelves (Figures 5F,F′,F″), was also diminished in the *Osr2-cre* ^*KI*^ *;Ctnnb1* ^*ex3f*^ palatal mesenchyme (Figures 5G,G′,G″,H). Surprisingly, the mesenchymal condensation in E13.5 *Osr2-cre* ^*KI*^ *;Ctnnb1* ^*ex3f*^ palatal shelves showed robust p-Smad1/5/8 staining (Figures 5G,G′,G″), which was only detected in the distal mesenchyme of the middle and the medial mesenchyme of the posterior WT palatal shelves (Figures 5F,F′,F″,H). Thus, the ectopic condensed mesenchyme in *Osr2-cre* ^*KI*^ *;Ctnnb1* ^*ex3f*^ palatal shelves was indicated to lose osteogenic capacity. Further immunofluorescence staining indicated that the ectopic condensed mesenchyme was devoid of Col1 staining but activated the fibrosis markers ɑ-SMA in the lateral condensation (Figures 5J,J′,J″). In contrast, E13.5 WT palatal shelves were devoid of ɑ-SMA staining, with widespread Col1 staining in the mesenchyme (Figures 5I,I′,I″,K). The FGF signaling indicators Etv4 and p-Erk1/2 showed little difference between E13.5 WT (Figures 5L,L′,L″) and *Osr2-cre* ^*KI*^ *;Ctnnb1* ^*ex3f*^ palatal shelves (Figures 5M,M′,M″,N). Therefore, these results strongly indicated that the condensed *Osr2-cre* ^*KI*^ *;Ctnnb1* ^*ex3f*^ palatal mesenchyme was deprived of osteogenic capacity and was most likely transformed into fibrogenic fate.

### The ectopic activation of BMP-inhibitors suppressed palatal osteogenesis

**FIGURE 6:**
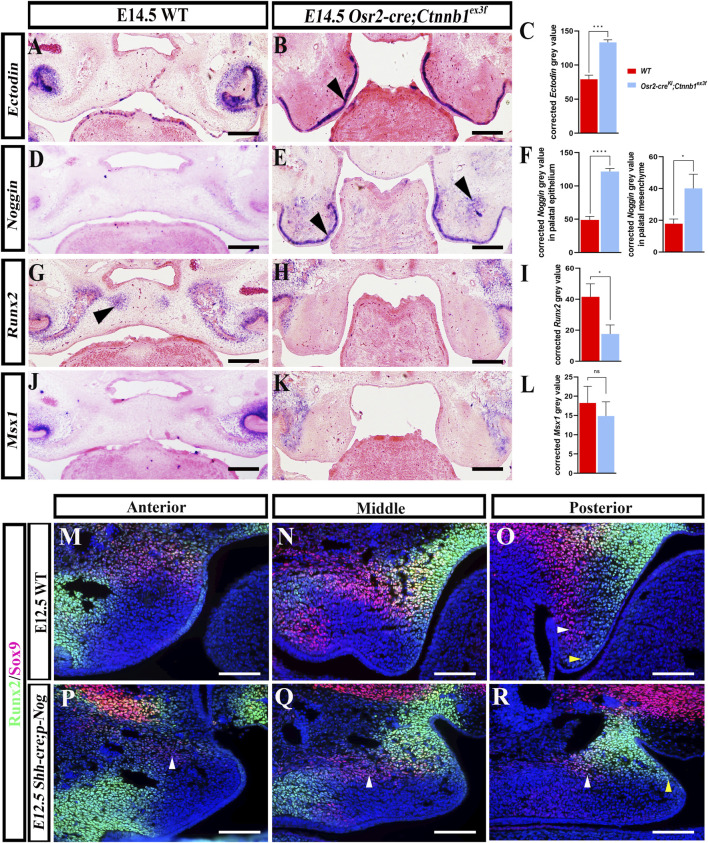
Ectopically activated BMP inhibitors in *Osr2-cre* ^*KI*^ *;Ctnnb1* ^*ex3f*^ palatal epithelium suppressed palatal osteogenesis. **(A–L)** *In situ* hybridization showed the *Ectodin* transcription in E14.5 WT **(A)** and *Osr2-cre* ^*KI*^ *;Ctnnb1* ^*ex3f*^ palatal shelves **(B)**. **(C)** Corrected *in situ* hybridization signal intensity of *Ectodin* showed the notable increase from 79.15 ± 6.03 in WT to 133.30 ± 3.73 (*p* < 0.001) in the *Osr2-cre* ^*KI*^ *;Ctnnb1* ^*ex3f*^ palatal epithelium; *Noggin* transcription in E14.5 WT **(D)** and *Osr2-cre* ^*KI*^ *;Ctnnb1* ^*ex3f*^ palatal shelves **(E)**. **(F)** Corrected *in situ* hybridization signal intensity of *Noggin* showed the significant increase from 48.94 ± 5.38 and 17.88 ± 2.92 in WT to 121.70 ± 4.42 (*p* < 0.0001) and 40.11 ± 8.91 (*p* < 0.05) in the *Osr2-cre* ^*KI*^ *;Ctnnb1* ^*ex3f*^ palatal epithelium and mesenchyme, respectively; *Runx2* transcription in E14.5 WT **(G)** and *Osr2-cre* ^*KI*^ *;Ctnnb1* ^*ex3f*^ palatal shelves **(H)**. **(I)** Corrected *in situ* hybridization signal intensity of *Runx2* markedly decreased from 41.59 ± 8.39 in WT to 17.63 ± 5.80 (*p* < 0.05) in *Osr2-cre* ^*KI*^ *;Ctnnb1* ^*ex3f*^ palatal mesenchyme; *Msx1* transcription in E14.5 WT **(J)** and *Osr2-cre* ^*KI*^ *;Ctnnb1* ^*ex3f*^ palatal shelves **(K)**. **(L)** Corrected *in situ* hybridization signal intensity of *Msx1* showed slight difference between WT (18.27 ± 4.31) and *Osr2-cre* ^*KI*^ *;Ctnnb1* ^*ex3f*^ palatal mesenchyme (14.86 ± 3.68, *p* > 0.05). **(M–R)** Immunofluorescence of Runx2 and Sox9 in E12.5 WT **(M–O)** and *Shh-cre;pMes-Noggin* palatal shelves **(P–R)**. Arrowheads indicate the positive signals of *in situ* hybridization; *, *p* < 0.05; ***, *p* < 0.001; ****, *p* < 0.0001; scale bars: 100 μm.

**FIGURE 7:**
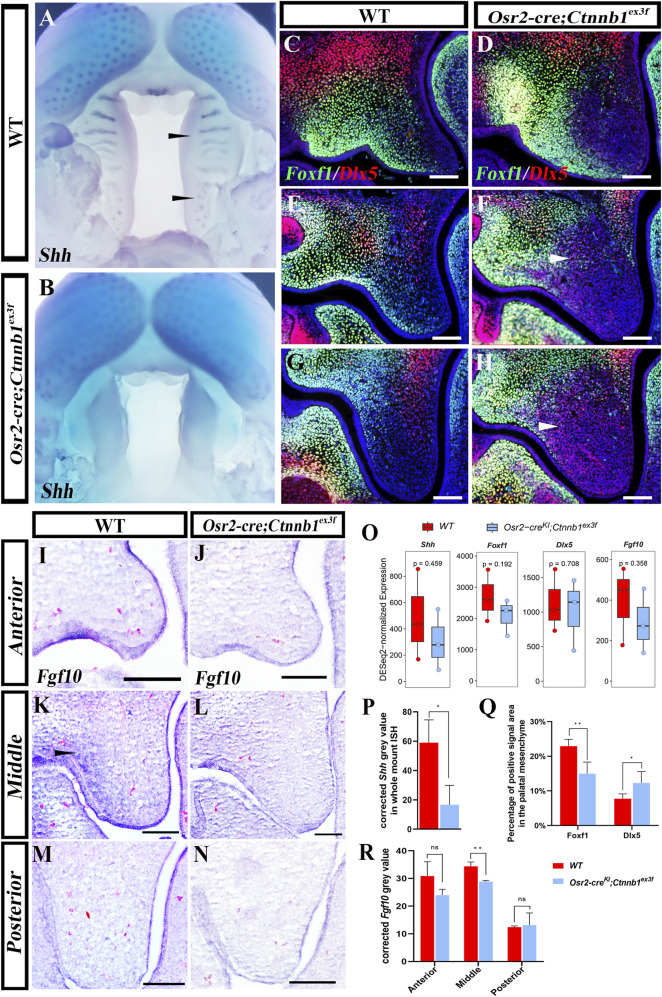
Suppressed *Shh* expression and related gene expression in *Osr2-cre* ^*KI*^ *;Ctnnb1* ^*ex3f*^ palatal shelves. **(A,B)** Whole-mount *in situ* hybridization indicated the *Shh* transcription in E13.5 WT rugae of the hard palate and knots of the soft palate (black arrowheads in **(A)**), while *Osr2-cre* ^*KI*^ *; Ctnnb1* ^*ex3f*^ palatal shelves were devoid of *Shh* expression **(B)**. **(C–H)** Immunofluorescence of Foxf1 and Dlx5 in E13.5 WT anterior **(C)**, middle **(E)**, and posterior **(G)** palatal shelves and in E13.5 *Ctnnb1* ^*ex3f*^ **(D)**, middle **(F)**, and posterior **(H)** palatal shelves. White arrowheads in **(F)** and **(H)** indicate the ectopic Dlx5 staining. **(I–N)** *In situ* hybridization of *Fgf10* in E13.5 WT anterior **(I)**, middle **(K)**, and posterior **(M)** palatal shelves and in E13.5 *Ctnnb1* ^*ex3f*^ **(J)** middle **(L)** and posterior **(N)** palatal shelves. **(O)** DESeq2-normalized *Shh*, *Foxf1*, *Dlx5*, and *Fgf10* expression from the bulk RNA-seq data showed no significant difference between WT and *Osr2-cre* ^*KI*^ *;Ctnnb1* ^*ex3f*^ palatal shelves. **(P)** Corrected signal intensity from the whole-mount *in situ* hybridization of *Shh* in E13.5 WT and *Osr2-cre* ^*KI*^ *;Ctnnb1* ^*ex3f*^ palatal shelves. **(Q)** Percentages of the Foxf1- and Dlx5-positive areas to the entire palatal shelves were markedly reduced and increased, respectively, in *Osr2-cre* ^*KI*^ *;Ctnnb1* ^*ex3f*^ palatal shelves compared to that in WT controls. **(R)** Corrected signal intensity from the *in situ* hybridization of *Fgf10* in E13.5 WT and *Osr2-cre* ^*KI*^ *;Ctnnb1* ^*ex3f*^ palatal shelves. (White arrowheads in **(F,H,K)** indicate the signals; *, *p* < 0.05; **, *p* < 0.01; scale bars: 100 μm).

To explore how persistent canonical Wnt signaling disrupted the osteogenic fate of the palatal mesenchyme, *in situ* hybridization was performed on E14.5 WT and *Osr2-cre* ^*KI*^ *;Ctnnb1* ^*ex3f*^ palates. The Wnt/BMP inhibitor *Ectodin* and the TGF-β/BMP inhibitor *Noggin*, both of which were excluded from the E14.5 WT palatal mesenchyme (Figures 6A,D), were ectopically activated in the *Osr2-cre* ^*KI*^ *;Ctnnb1* ^*ex3f*^ palatal epithelium (Figures 6B,C) and in both the *Osr2-cre* ^*KI*^ *;Ctnnb1* ^*ex3f*^ palatal epithelium and condensed mesenchyme (Figures 6E,F), respectively. *Runx2* transcription indicated the secondary ossification centers in the E14.5 WT palates (Figure 6G), while it displayed no transcripts in the *Osr2-cre* ^*KI*^ *;Ctnnb1* ^*ex3f*^ palatal shelves (Figures 6H,I). *Msx1* transcription was detected in neither the E14.5 WT (Figure 6J) nor the *Osr2-cre* ^*KI*^ *;Ctnnb1* ^*ex3f*^ palates (Figures 6K,L). These results indicated impaired osteogenesis in *Osr2-cre* ^*KI*^ *;Ctnnb1* ^*ex3f*^ palates correlated with the ectopic activation of *Ectodin* and *Noggin*. To verify the repression of epithelial-derived Noggin on the osteogenic specification of the palatal mesenchyme, we studied *Runx2* and *Sox9* expression in the palatal shelves of *Shh-cre;pMes-Noggin* mice. Immunofluorescence staining showed significantly reduced Runx2 distribution, especially in the posterior shelf, and the almost absent Sox9 domain in the *Shh-cre;pMes-Noggin* palatal shelves (Figures 7M–O) compared to that in the E12.5 WT controls (Figures 7M–O), which indicated the suppression of osteogenic specification of palatal mesenchyme by epithelial-derived Noggin.

### Diminished *Shh* expression and disrupted mediolateral patterning in *Osr2-cre* ^*KI*^ *;Ctnnb1* ^*ex3f*^ palatal shelves

To further understand the impacts of persistent canonical Wnt signaling on palatogenesis, *Shh* expression and SHH signaling were examined. Whole-mount *in situ* hybridization revealed the abrogation of *Shh* transcription in E13.5 *Osr2-cre* ^*KI*^ *;Ctnnb1* ^*ex3f*^ palatal epithelium (Figure 7B), which is in contrast to the specific restriction in WT rugae (Figure 7A). Consistently, the immunofluorescence of Foxf1, a downstream target of mesenchymal SHH signaling, which was distributed in the lateral side of the E13.5 WT palatal mesenchyme (Figures 7C,E,G), was reduced to the proximal side in the middle and posterior *Osr2-cre* ^*KI*^ *;Ctnnb1* ^*ex3f*^ palatal mesenchyme, though it had little impact in the anterior side (Figures 7D,F,H). In contrast, the immunofluorescence of Dlx5, which marked the medial side of the palatal mesenchyme (Figures 7C,E,G), extended to the lateral mesenchyme in the middle and posterior *Osr2-cre* ^*KI*^ *;Ctnnb1* ^*ex3f*^ palatal mesenchyme (Figures 7D,F,H). *In situ* hybridization indicated that another downstream target of mesenchymal SHH signaling, *Fgf10*, which was activated in the lateral mesenchyme of middle palatal shelves (Figures 7I,K,M), had noticeably faded in E13.5 *Osr2-cre* ^*KI*^ *;Ctnnb1* ^*ex3f*^ palatal mesenchyme (Figures 7J,L,N). Although the DESeq2-normalized expression of *Shh*, *Foxf1*, *Dlx5*, and *Fgf10* showed no difference when comparing bulk RNA-seq data of the E13.5 WT and *Osr2-cre* ^*KI*^ *;Ctnnb1* ^*ex3f*^ palatal shelves (Figure 7O), the quantified signal intensity by ImageJ verified the reduced transcription of the *Shh*, *Fgf10*, and Foxf1 domains (Figures 7P–R), along with the increased Dlx5 domain in E13.5 *Osr2-cre* ^*KI*^ *; Ctnnb1* ^*ex3f*^ palatal shelves (Figure 7R), which indicated that the persistent canonical Wnt activity was most likely important for the suppressed *Shh* transcription and SHH signaling and the disrupted mediolateral patterning in the palatal shelf.

## Discussion

The cleft palates in *Osr2-cre* ^*KI*^ *;Ctnnb1* ^*f/f*^ and *Osr2-cre* ^*KI*^ *;Ctnnb1* ^*ex3f*^ mice have been reported previously (Chen et al., 2009; Janečková et al., 2023); however, how persistent canonical Wnt activity in the palatal mesenchyme causes cleft palates remains elusive. In this study, we explicated a series of defects in *Osr2-cre* ^*KI*^ *;Ctnnb1* ^*ex3f*^ palatogenesis, including the failed elevation of palatal shelves, ectopic condensed palatal mesenchyme, impaired palatal osteogenesis, and agenesis of the soft palate. Unlike the cleft soft palates in *Wnt1-cre;Fam20b* ^*f/f*^ or *Osr2-cre* ^*KI*^ *;pMes-Noggin* mice (Deng et al., 2021; Li et al., 2021; Chen et al., 2023), the soft palatogenesis in *Osr2-cre* ^*KI*^ *;Ctnnb1* ^*ex3f*^ mice was never initiated, which provided an ideal tool to study the genesis of soft palates. We integrated the posteriorly extended *Wnt5a*-expressing domain, the ectopic condensed mesenchyme, and the agenesis of soft palates into a comprehensive interpretation. The constitutively activated canonical Wnt signaling in palatal mesenchymal cells was indicated to extend the anterior *Wnt5a* expression into the middle of the palatal mesenchyme. Since Wnt5a acted as a chemokine and induced palatal mesenchymal migration along the posterior-to-anterior orientation (He et al., 2008), the increased and extended *Wnt5a* transcription most likely enhanced the posterior–anterior migration in *Osr2-cre* ^*KI*^ *;Ctnnb1* ^*ex3f*^ palatal shelves. The increased cell density and anteriorly extended Tbx15 expression from WT soft palates with Wnt5a supplement supported the enhanced posterior-to-anterior migration. With more posterior mesenchymal cells, including the mesenchymal cells for the presumptive soft palates, migrating toward and condensing in the anterior palates, the genesis of soft palates was indicated to be disabled. Furthermore, a recent study utilizing *Sox9-cre* ^*ERT*^ *;Ctnnb1* ^*ex3f*^ mice also showed cleft palates with the condensed mesenchyme, along with increased ɑ-actin-1/4 and F-actin, indicating a model in which upregulated cell adhesion was caused due to canonical Wnt signaling (Wang et al., 2022), which was consistent with the highly increased integrin ɑv in *Osr2-cre* ^*KI*^ *;Ctnnb1* ^*ex3f*^ palatal shelves. These consequences indicated that the ectopic mesenchymal condensation in *Osr2-cre* ^*KI*^ *;Ctnnb1* ^*ex3f*^ palatal shelves most likely originated from the anteriorly migrated palatal mesenchymal cells condensed by the enhanced cell adhesion.

As a non-canonical Wnt ligand, Wnt5a has been proven to promote cell migration and fibrosis by enhancing *Sfrp5* transcription (Chatani et al., 2015; Zou et al., 2021; Trinh-Minh et al., 2024), which coincided with the increased ɑ-SMA-expressing domain and *Sfrp5* expression in *Osr2-cre* ^*KI*^ *;Ctnnb1* ^*ex3f*^ palatal shelves. Further analysis revealed that although canonical Wnt signaling would be antagonized by Wnt5a in tumor cells (Yuzugullu et al., 2009), Wnt5a activates or suppresses canonical Wnt activity depending on the receptor context (Mikels and Nusse, 2006). However, the effects of canonical Wnt activity on the non-canonical Wnt signaling or *Wnt5a* expression are still unknown. Our study indicated the activation of *Wnt5a* due to persistent canonical Wnt activity in the palatal mesenchyme, which requires further exploration of the molecular regulation. Notably, the enhanced *Wnt5a* transcription in the *Osr2-cre* ^*KI*^ *;Ctnnb1* ^*ex3f*^ middle palatal shelves was excluded from the DEGs of bulk RNA-seq, most likely due to the local altered gene expression being shared by the entire palatal shelves.

The impaired palatal osteogenesis in *Osr2-cre* ^*KI*^ *;Ctnnb1* ^*ex3f*^ palatal shelves could also be attributed to persistent canonical Wnt activity. Although Wnt5a was reported to enhance canonical Wnt signaling during osteoblastogenesis (Okamoto et al., 2014), the secreted Wnt inhibitor, Sfrp2, could promote osteogenic differentiation by antagonizing canonical Wnt signaling (Jin et al., 2017; Wen et al., 2020; Yang et al., 2020; Wang et al., 2021; Akova Ölken et al., 2022). Thus, the diminished *Sfrp2* expression in *Osr2-cre* ^*KI*^ *;Ctnnb1* ^*ex3f*^ palatal mesenchyme strongly indicated the association of persistent canonical Wnt activity with impaired palatal osteogenesis by suppressing *Sfrp2* transcription. Meanwhile, the ectopic activated *Ectodin* and *Noggin* in *Osr2-cre* ^*KI*^ *;Ctnnb1* ^*ex3f*^ palatal epithelium was also implicated in depriving the underlying mesenchymal cells of the presumptive palatal bones of osteogenic capacity. Our previous study had reported that the overexpression of *Noggin* in the palatal mesenchyme strongly suppressed palatal osteogenesis (Li et al., 2021). Furthermore, in this study, overexpressed *Noggin* in the palatal epithelium was also associated with disrupted osteogenic commitment of the palatal mesenchyme, which supported the impaired palatal osteogenesis caused by the epithelium-derived Noggin or Ectodin. Such ectopic activation of *Noggin* by the canonical Wnt activity in adjacent tissues has been reported in our previous study on tooth development (Chen et al., 2019) and the recent study on muscle stromal progenitors (Kajabadi et al., 2023), though the reciprocal interactions between the canonical Wnt activity-processing tissue and the *Noggin*-expressing tissue still require exploration. Moreover, in *Osr2-cre* ^*KI*^ *;Ctnnb1* ^*ex3f*^ palatal condensation, a series of osteogenic markers, namely, Runx2, Osx, and ColI, were significantly reduced, but the fibrosis/dermal markers Tbx15 and ɑ-SMA were activated, indicating a transition of osteogenic fate of palatal mesenchyme into fibrosis/dermal specification.

Eventually, the disrupted mediolateral patterning of palatal shelves was also associated with the persistent canonical Wnt activity. *Shh* and *Fgf10*, activated in the lateral epithelium and mesenchyme, respectively, of the middle palatal shelves, and Dlx5, p-Smad1/5/8, and Sox9 in the medial mesenchyme were regarded as hallmarks of the lateral–medial patterning of palatal shelves (Lan et al., 2004; Han et al., 2009; He et al., 2010). In *Osr2-cre* ^*KI*^ *;Ctnnb1* ^*ex3f*^ palatal shelves, the diminished *Shh, Foxf1*, and *Fgf10* expression indicated a loss of lateral identity, while the medial-to-lateral extension of Dlx5, Runx2, and p-Smad1/5/8 domain in the condensed *Osr2-cre* ^*KI*^ *;Ctnnb1* ^*ex3f*^ palatal mesenchyme indicated an expansion of medial identity. Although the Sox9 domain was also diminished throughout the *Osr2-cre* ^*KI*^ *;Ctnnb1* ^*ex3f*^ palatal shelves, it could be interpreted as the loss of osteogenic fate of the medial mesenchyme instead of the loss of medial identity because Sox9 marked the chondro-osteogenic progenitors of craniofacial mesenchyme (Akiyama et al., 2002; Mori-Akiyama et al., 2003; Dash and Trainor, 2020). Thus, the lateral–medial patterning of *Osr2-cre* ^*KI*^ *;Ctnnb1* ^*ex3f*^ palatal shelves was indicated to be interrupted by the persistent canonical Wnt activity. Combined with the lateral–medial patterning of many other ECM in palatal shelves (Chiquet et al., 2016; Wang et al., 2020), it indicated that the lateral–medial patterning of palatal shelves contributed to palatal elevation through the ECM pattern. However, whether and how the lateral–medial patterning of palatal shelves contributes to palatal elevation is still unknown. Our previous study reported cleft palates in *Osr2-cre* ^*KI*^ *;Rosa26R-Fgf8* mice associated with the disrupted lateral–medial axis of the palatal shelves despite increased cell proliferation in the palatal mesenchyme (Wu et al., 2015). Although the previous study indeed demonstrated a posterior-to-anterior migration of palatal mesenchymal cells along the Wnt5a gradient (He et al., 2008), to date, there is no convincing evidence supporting the contribution of cell migration to palatal elevation.

In summary, our present study reported a disabled genesis of the soft palate with failed osteogenesis and mediolateral patterning in the palatal shelves when canonical Wnt signaling was constitutively activated in the palatal mesenchyme. An ectopic *Wnt5a* extension by the persistent canonical Wnt signaling was associated with the extra-anterior migration of posterior mesenchymal cells into the condensed mesenchyme, which most likely impaired the genesis of soft palates. Moreover, the persistent canonical Wnt activity appears to deprive the palatal mesenchyme of osteogenic capacity that was correlated to the suppressed *Sfrp2* expression, ectopically activated *Noggin* and *Ectodin* in the palatal epithelium, and even the transformation of the osteogenic fate into fibrosis/dermal specification. Meanwhile, the mediolateral pattering of palatal shelves was also indicated to be disrupted by the persistent canonical Wnt activity in the palatal mesenchyme.

## Data Availability

The original contributions presented in the study are included in the article/Supplementary Material; further inquiries can be directed to the corresponding authors.

## Supplementary material

SUPPLEMENTARY FIGURE S1 Cryostat section of the palates in *Osr2-cre* ^*KI*^ *; Rosa26R-mT/mG* mice. **(A)** Middle palatal shelves of E13.5 *Osr2-cre* ^*KI*^ *; Rosa26R-mT/mG* mice. **(B)** Middle hard palate of E16.5 *Osr2-cre* ^*KI*^ *; Rosa26R-mT/mG* mice. **(C)** Posterior hard palate of E16.5 *Osr2-cre* ^*KI*^ *; Rosa26R-mT/mG* mice. **(D)** Soft palate of E16.5 *Osr2-cre* ^*KI*^ *;Rosa26R-mT/mG* mice.

SUPPLEMENTARY FIGURE S2 BrdU labeling and TUNEL assays in the palates of *Osr2-cre* ^*KI*^ *;Ctnnb1* ^*ex3f*^ mice. **(A)** Most anterior palate in front of mesenchymal condensation in E13.5 *Osr2-cre* ^*KI*^ *;Ctnnb1* ^*ex3f*^ mice. **(B)** Anterior palate with condensed mesenchyme in E13.5 *Osr2-cre* ^*KI*^ *;Ctnnb1* ^*ex3f*^ mice. **(C-H)** TUNEL assay of the palates in E13.5 *Osr2-cre* ^*KI*^ *;Ctnnb1* ^*ex3f*^ mice. Scale bars: 100 μm.

SUPPLEMENTARY DATA SHEET 1 DEGs found by comparing the gene expression profile of E13.5 *Osr2-cre* ^*KI*^ *;Ctnnb1* ^*ex3f*^ palates with that of WT palates.

SUPPLEMENTARY DATA SHEET 2 Enriched functions and signaling pathways via GO analysis of the DEGs by comparing the gene expression profile of E13.5 *Osr2-cre* ^*KI*^ *;Ctnnb1* ^*ex3f*^ palates with that of WT palates.

## References

[B1] Akiyama H. Chaboissier M. C. Martin J. F. Schedl A. de Crombrugghe B. (2002). The transcription factor Sox9 has essential roles in successive steps of the chondrocyte differentiation pathway and is required for expression of Sox5 and Sox6. Genes Dev. 16, 2813–2828. 10.1101/gad.1017802 12414734 PMC187468

[B2] Akova Ölken E. Aszodi A. Taipaleenmäki H. Saito H. Schönitzer V. Chaloupka M. (2022). SFRP2 overexpression induces an osteoblast-like phenotype in prostate cancer cells. Cells 11, 4081. 10.3390/cells11244081 36552843 PMC9777425

[B3] Bush J. O. Jiang R. (2016). Palatogenesis: morphogenetic and molecular mechanisms of secondary palate development. Development 139, 231–243. 10.1242/dev.067082 22186724 PMC3243091

[B4] Chatani N. Kamada Y. Kizu T. Ogura S. Furuta K. Egawa M. (2015). Secreted frizzled-related protein 5 (Sfrp5) decreases hepatic stellate cell activation and liver fibrosis. Liver Int. 35, 2017–2026. 10.1111/liv.12757 25488180

[B5] Chen J. Lan Y. Baek J. A. Gao Y. Jiang R. (2009). Wnt/beta-catenin signaling plays an essential role in activation of odontogenic mesenchyme during early tooth development. Dev. Biol. 334, 174–185. 10.1016/j.ydbio.2009.07.015 19631205 PMC2752344

[B6] Chen X. Liu J. Li N. Wang Y. Zhou N. Zhu L. (2019). Mesenchymal Wnt/beta-catenin signaling induces Wnt and BMP antagonists in dental epithelium. Organogenesis 15, 55–67. 10.1080/15476278.2019.1633871 31240991 PMC6668654

[B7] Chen X. Li N. Hu P. Li L. Li D. Liu H. (2023). Deficiency of Fam20b-Catalyzed glycosaminoglycan chain synthesis in neural crest leads to cleft palate. Int. J. Mol. Sci. 24, 9634. 10.3390/ijms24119634 37298583 PMC10253313

[B8] Chen X. Liu H. Huang Y. Li L. Jiang X. Liu B. (2025). FAM20B-Catalyzed glycosylation regulates the chondrogenic and osteogenic differentiation of the embryonic condyle by controlling IHH diffusion and release. Int. J. Mol. Sci. 26, 4033. 10.3390/ijms26094033 40362273 PMC12071210

[B9] Chiquet B. T. Blanton S. H. Burt A. Ma D. Stal S. Mulliken J. B. (2008). Variation in WNT genes is associated with non-syndromic cleft lip with or without cleft palate. Hum. Mol. Genet. 17, 2212–2218. 10.1093/hmg/ddn121 18413325 PMC2852032

[B10] Chiquet M. Blumer S. Angelini M. Mitsiadis T. A. Katsaros C. (2016). Mesenchymal remodeling during palatal shelf elevation revealed by extracellular matrix and F-Actin expression patterns. Front. Physiol. 7, 392. 10.3389/fphys.2016.00392 27656150 PMC5013070

[B11] Dash S. Trainor P. A. (2020). The development, patterning and evolution of neural crest cell differentiation into cartilage and bone. Bone 137, 115409. 10.1016/j.bone.2020.115409 32417535

[B12] Deng J. Wang S. Li N. Chen X. Wang B. Liu H. (2021). Noggin overexpression impairs the development of muscles, tendons, and aponeurosis in soft palates by disrupting BMP-smad and Shh-Gli1 signaling. Front. Cell Dev. Biol. 9, 711334. 10.3389/fcell.2021.711334 34557486 PMC8453081

[B13] Han J. Mayo J. Xu X. Li J. Bringas P. Jr. Maas R. L. (2009). Indirect modulation of Shh signaling by Dlx5 affects the oral-nasal patterning of palate and rescues cleft palate in Msx1-null mice. Development 136, 4225–4233. 10.1242/dev.036723 19934017 PMC2781056

[B14] He F. Xiong W. Yu X. Espinoza-Lewis R. Liu C. Gu S. (2008). Wnt5a regulates directional cell migration and cell proliferation via Ror2-mediated noncanonical pathway in mammalian palate development. Development 135, 3871–3879. 10.1242/dev.025767 18948417 PMC3010758

[B15] He F. Xiong W. Wang Y. Matsui M. Yu X. Chai Y. (2010). Modulation of BMP signaling by Noggin is required for the maintenance of palatal epithelial integrity during palatogenesis. Dev. Biol. 347, 109–121. 10.1016/j.ydbio.2010.08.014 20727875 PMC3010875

[B16] He F. Xiong W. Wang Y. Li L. Liu C. Yamagami T. (2011). Epithelial Wnt/β-catenin signaling regulates palatal shelf fusion through regulation of Tgfβ3 expression. Dev. Biol. 350, 511–519. 10.1016/j.ydbio.2010.12.021 21185284 PMC3040240

[B17] Janečková E. Feng J. Li J. Rodriguez G. Chai Y. (2019). Dynamic activation of Wnt, Fgf, and Hh signaling during soft palate development. PLoS One 14, e0223879. 10.1371/journal.pone.0223879 31613912 PMC6793855

[B18] Janečková E. Feng J. Guo T. Han X. Ghobadi A. Araujo-Villalba A. (2023). Canonical Wnt signaling regulates soft palate development by mediating ciliary homeostasis. Development 150, dev201189. 10.1242/dev.201189 36825984 PMC10108707

[B19] Jin L. Cao Y. Yu G. Wang J. Lin X. Ge L. (2017). SFRP2 enhances the osteogenic differentiation of apical papilla stem cells by antagonizing the canonical WNT pathway. Cell Mol. Biol. Lett. 22, 14. 10.1186/s11658-017-0044-2 28794794 PMC5547503

[B20] Kajabadi N. Low M. Jacques E. Lad H. Tung L. W. Babaeijandaghi F. (2023). Activation of beta-catenin in mesenchymal progenitors leads to muscle mass loss. Dev. Cell 58, 489–505.e7. 10.1016/j.devcel.2023.02.009 36898377

[B21] Lan Y. Ovitt C. E. Cho E. S. Maltby K. M. Wang Q. Jiang R. (2004). Odd-skipped related 2 (Osr2) encodes a key intrinsic regulator of secondary palate growth and morphogenesis. Development 131, 3207–3216. 10.1242/dev.01175 15175245

[B22] Li C. Lan Y. Jiang R. (2017a). Molecular and cellular mechanisms of palate development. J. Dent. Res. 96, 1184–1191. 10.1177/0022034517703580 28745929 PMC5613875

[B23] Li C. Lan Y. Krumlauf R. Jiang R. (2017b). Modulating wnt signaling rescues palate morphogenesis in Pax9 mutant mice. J. Dent. Res. 96, 1273–1281. 10.1177/0022034517719865 28692808 PMC5613879

[B24] Li J. Rodriguez G. Han X. Janečková E. Kahng S. Song B. (2019). Regulatory mechanisms of soft palate development and malformations. J. Dent. Res. 98, 959–967. 10.1177/0022034519851786 31150594 PMC6651766

[B25] Li N. Liu J. Liu H. Wang S. Hu P. Zhou H. (2021). Altered BMP-Smad4 signaling causes complete cleft palate by disturbing osteogenesis in palatal mesenchyme. J. Mol. Histol. 52, 45–61. 10.1007/s10735-020-09922-4 33159638

[B26] Liu Y. Wang M. Zhao W. Yuan X. Yang X. Li Y. (2015). Gpr177-mediated wnt signaling is required for secondary palate development. J. Dent. Res. 94, 961–967. 10.1177/0022034515583532 25922332

[B27] Liu C. Zhou N. Li N. Xu T. Chen X. Zhou H. (2022). Disrupted tenogenesis in masseter as a potential cause of micrognathia. Int. J. Oral. Sci. 14, 50. 10.1038/s41368-022-00196-y 36257937 PMC9579150

[B28] Mikels A. J. Nusse R. (2006). Purified Wnt5a protein activates or inhibits beta-catenin-TCF signaling depending on receptor context. PLoS Biol. 4, e115. 10.1371/journal.pbio.0040115 16602827 PMC1420652

[B29] Mori-Akiyama Y. Akiyama H. Rowitch D. H. de Crombrugghe B. (2003). Sox9 is required for determination of the chondrogenic cell lineage in the cranial neural crest. Proc. Natl. Acad. Sci. USA. 100, 9360–9365. 10.1073/pnas.1631288100 12878728 PMC170923

[B30] Okamoto M. Udagawa N. Uehara S. Maeda K. Yamashita T. Nakamichi Y. (2014). Noncanonical Wnt5a enhances Wnt/beta-catenin signaling during osteoblastogenesis. Sci. Rep. 4, 4493. 10.1038/srep04493 24670389 PMC3967152

[B31] Perez-Moreno M. Fuchs E. (2006). Catenins: keeping cells from getting their signals crossed. Dev. Cell. 11, 601–612. 10.1016/j.devcel.2006.10.010 17084354 PMC2405914

[B32] Reynolds K. Kumari P. Rincon L. S. Gu R. Ji Y. Kumar S. (2019). Wnt signaling in orofacial clefts: crosstalk, pathogenesis and models. Dis. Model. Mech. 12, dmm037051. 10.1242/dmm.037051 30760477 PMC6398499

[B33] Salinas P. C. (2007). Modulation of the microtubule cytoskeleton: a role for a divergent canonical Wnt pathway. Trends. Cell. Biol. 17, 333–342. 10.1016/j.tcb.2007.07.003 17643305

[B34] Trinh-Minh T. Chen C. W. Tran Manh C. Li Y. N. Zhu H. Zhou X. (2024). Noncanonical WNT5A controls the activation of latent TGF-beta to drive fibroblast activation and tissue fibrosis. J. Clin. Invest. 134, e159884. 10.1172/JCI159884 38747285 PMC11093613

[B35] Wang X. Li C. Zhu Z. Yuan L. Chan W. Y. Sha O. (2020). Extracellular matrix remodeling during palate development. Organogenesis 16, 43–60. 10.1080/15476278.2020.1735239 32233728 PMC7531623

[B36] Wang C. Wang Y. Wang H. Yang H. Cao Y. Xia D. (2021). SFRP2 enhances dental pulp stem cell-mediated dentin regeneration in rabbit jaw. Oral Dis. 27, 1738–1746. 10.1111/odi.13698 33128313

[B37] Wang X. Liu W. Luo X. Zheng Q. Shi B. Liu R. (2022). Mesenchymal β-catenin signaling affects palatogenesis by regulating α-actinin-4 and F-actin. Oral Dis. 29, 3493–3502. 10.1111/odi.14408 36251469

[B38] Wen Q. Jing J. Han X. Feng J. Yuan Y. Ma Y. (2020). Runx2 regulates mouse tooth root development Via activation of WNT inhibitor NOTUM. J. Bone Min. Res. 35, 2252–2264. 10.1002/jbmr.4120 32569388 PMC7689689

[B39] Wu W. Gu S. Sun C. He W. Xie X. Li X. (2015). Altered FGF signaling pathways impair cell proliferation and elevation of palate shelves. PLoS One 10, e0136951. 10.1371/journal.pone.0136951 26332583 PMC4558018

[B40] Yang H. Li G. Han N. Zhang X. Cao Y. Cao Y. (2020). Secreted frizzled-related protein 2 promotes the osteo/odontogenic differentiation and paracrine potentials of stem cells from apical papilla under inflammation and hypoxia conditions. Cell Prolif. 53, e12694. 10.1111/cpr.12694 31568642 PMC6985663

[B41] Yuzugullu H. Benhaj K. Ozturk N. Senturk S. Celik E. Toylu A. (2009). Canonical Wnt signaling is antagonized by noncanonical Wnt5a in hepatocellular carcinoma cells. Mol. Cancer 8, 90. 10.1186/1476-4598-8-90 19849855 PMC2770486

[B42] Zhang Z. Pan X. Chen M. Bai M. (2022). Wnt signalling in oral and maxillofacial diseases. Cell Biol. Int. 46, 34–45. 10.1002/cbin.11708 34643311

[B43] Zou Y. Pan L. Shen Y. Wang X. Huang C. Wang H. (2021). Cardiac Wnt5a and Wnt11 promote fibrosis by the crosstalk of FZD5 and EGFR signaling under pressure overload. Cell Death Dis. 12, 877. 10.1038/s41419-021-04152-2 34564708 PMC8464604

