# Supplementary figures and images for: Persistent Wnt/β-catenin signaling disables soft palatogenesis and palatal osteogenesis by inducing mesenchymal condensation
Source: Front Cell Dev Biol. 2026 Mar 20;14:1740081. doi: 10.3389/fcell.2026.1740081 (PMC13047078; doi:10.3389/fcell.2026.1740081)

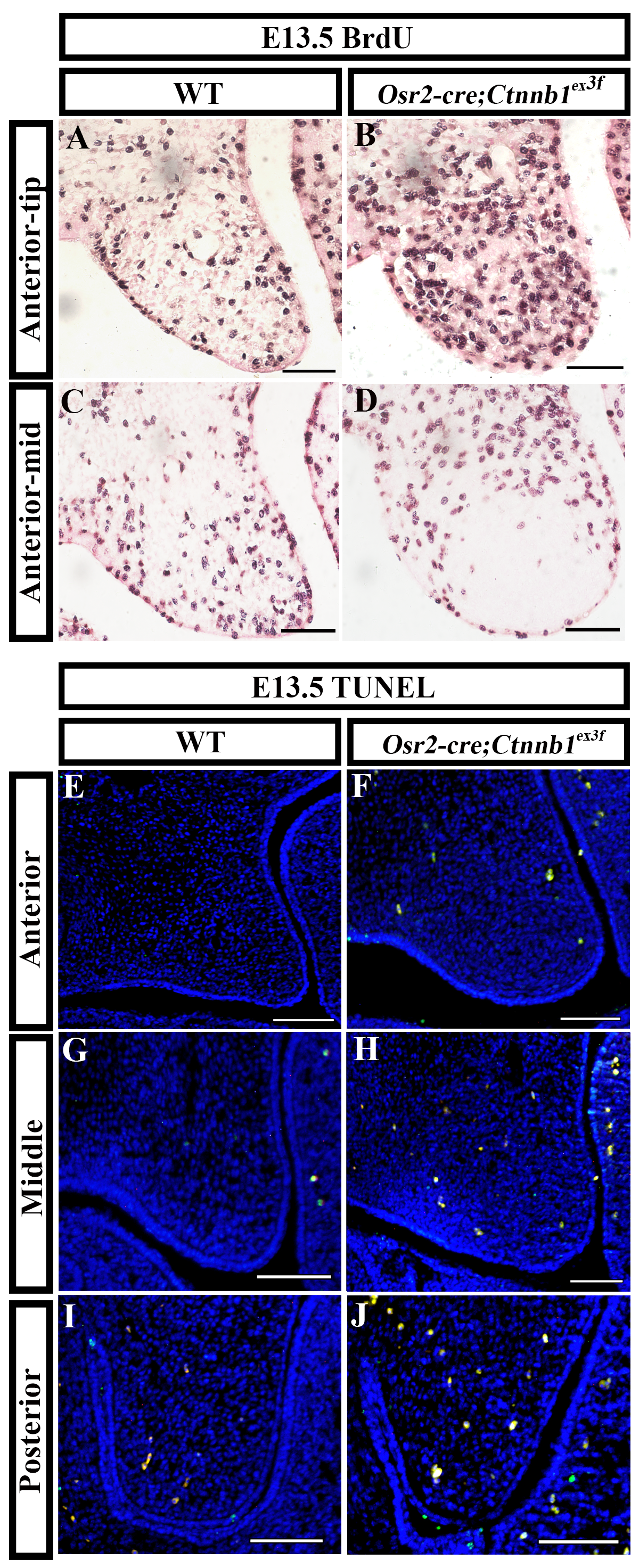

Supplement: Supplementary file 1 [file Image2.tif]

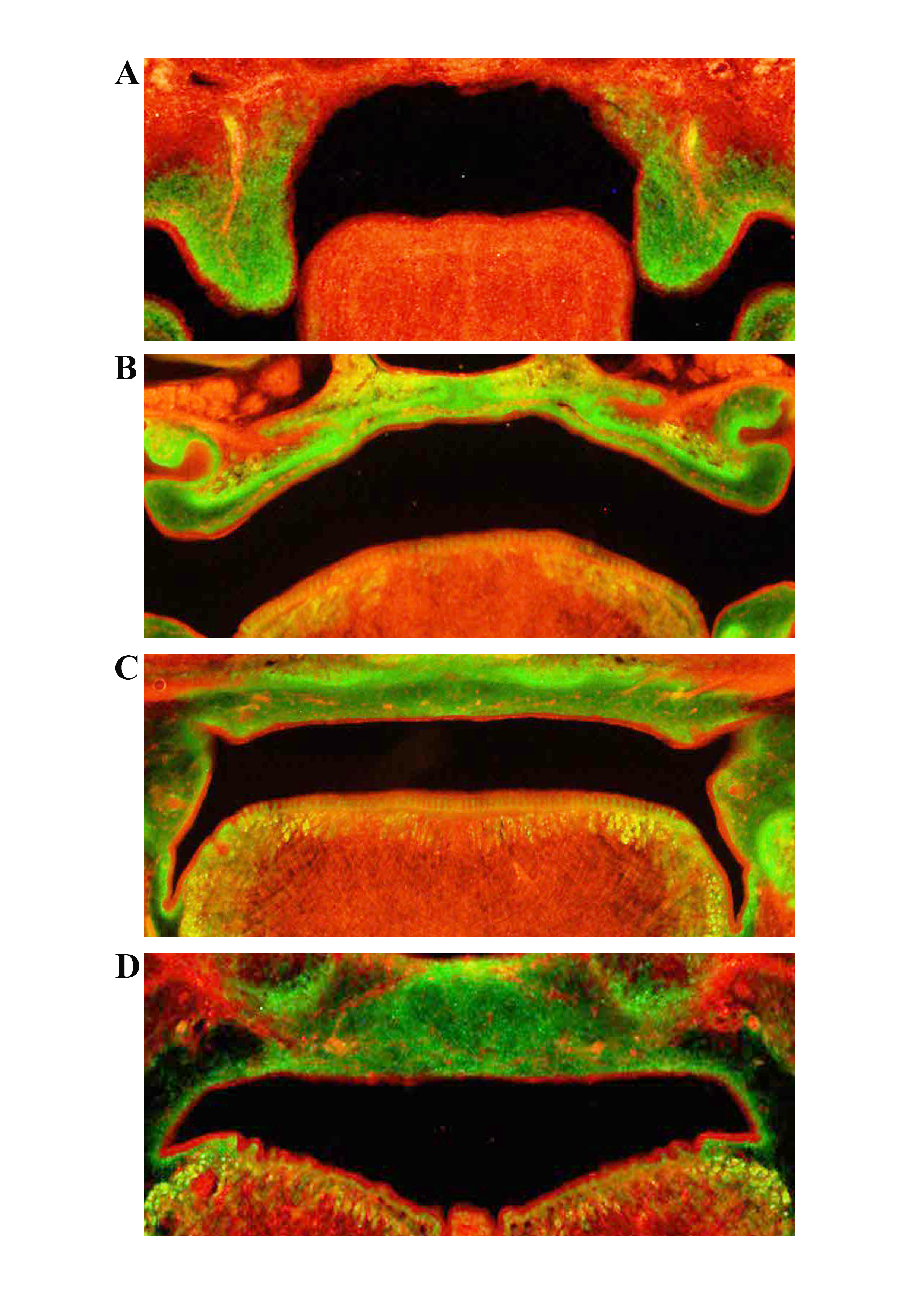

Supplement: Supplementary file 2 [file Image1.tif]
